# The Role of Parenting Styles on Behavior Problem Profiles of Adolescents

**DOI:** 10.3390/ijerph16152767

**Published:** 2019-08-02

**Authors:** Bárbara Lorence, Victoria Hidalgo, Javier Pérez-Padilla, Susana Menéndez

**Affiliations:** 1 Faculty of Psychology, University of Seville, Calle Camilo José Cela, s/n, 41018 Sevilla, Spain; 2 Faculty of Humanities and Education Sciences, University of Jaen, Campus Las Lagunillas, s/n, 23071 Jaén, Spain; 3 Faculty of Education, Psychology and Sports Sciences, University of Huelva, Campus El Carmen, Avenida de las Fuerzas Armadas, s/n, 21007 Huelva, Spain

**Keywords:** parenting styles, adolescent, behavior problems, stressful life events

## Abstract

Parental behavior is one of the most influential factors on the development of adolescent externalizing and internalizing behavior problems. These behavioral problems are closely related and often co-occur. The objectives of this work were: (i) to identify adolescents profiles according to their behavior problems; (ii) to explore individual, family, and social characteristics associated with these profiles; and (iii) to analyze the potential role of parenting styles in belonging to adolescents’ profiles. A total of 449 Spanish adolescents (223 from families declared at-risk and enrolled in Child Welfare Services and 226 from families from the general population) participated in this study. The analyses revealed three profiles of adolescents based on external and internal behavior problems (adjusted, external maladjustment, and internal maladjustment). Parenting styles explained the adolescents’ belonging to different profiles, in which the indulgent style was the most favorable in general terms. The distinctive role of parenting styles on two types of maladjustment profiles was confirmed. The relationship between parenting styles and adolescent adjustment is a key component that should be included in interventions according to adolescents’ behavior problem profiles. Furthermore, the results shed light on the need that family interventions are complemented with individualized interventions with adolescents that accumulate stressful life events.

## 1. Introduction

There is broad consensus that the goal of parenting is to establish positive relationships with children and adolescents within the family to ensure their development and well-being. The contemporary concept of positive parenting [1] implies that parent–child relationships should be based on affection, support, communication, stimulation, and structuring in routines, in the establishment of limits, norms, and consequences, as well as in the involvement in the daily life of children and adolescents [2]. However, positive parenting is a difficult task, especially during adolescence, when there is a tendency toward an increase in family conflict, which is due in part to developmental changes and new challenges faced by boys and girls [3,4]. In view of this reality, current family support policies in most countries provide parental education programs for the parents of adolescents [1]. In these interventions, parenting styles are often a central component in promoting positive parenting. This paper explores the relationship between parenting styles and adolescent adjustment profiles, with the overall aim of identifying key components of parenting styles for preventing internalizing and externalizing problems.

### 1.1. Psychosocial Adjustment in Adolescence: Behavior Problems and Self-Concept

Normative changes that occur during adolescence have been related to an increase in self-perceived maladjustment during this developmental stage. Psychological adjustment in adolescence is explained by the interaction of the multiple changes that take place at these ages [5,6]. In terms of self-development, there tends to be a drop in boys’ and girls’ assessment of themselves during early adolescence, with this gradually recovering as they reach adulthood [7]. Self-concept is an indicator of adjustment associated with behavior problems [8]. These types of problems affect many adolescents [9], although few persist with behavioral problems into adulthood [10,11]. There is an increase in externalizing problems, such as delinquency, substance abuse, aggressiveness, or breaking of rules [12,13]; and internal problems related to social isolation, withdrawal, anxiety, and depression [14,15]. It has been found that the prevalence of certain disorders follows different patterns in males and females. Most of the studies that make sex comparisons highlight a higher rate of internalizing problems in girls [16,17] and a higher rate of externalizing problems in boys [6,17,18,19], although not all recent studies found such a clear-cut difference along sex lines [20,21]. The co-occurrence, which is referred to the simultaneous presence of both internalizing and externalizing problems, has also come under close scrutiny in adolescent studies [9,22]. The previous research goals tend to focus exclusively on internalizing or externalizing problems, without taking into account the development of co-occurring symptoms for some children. The studies of the etiology or treatment of internalizing and externalizing symptoms in adolescents should account for co-occurrence (e.g., identifying existing adolescent adjustment profiles).

In addition to normative changes, several studies report a strong association between the accumulation of stressful life events in adolescents and both internalizing and externalizing problems, regardless of sex [23,24,25,26]. The accumulative risk hypothesis understands that experiencing stressful circumstances has a negative impact on people’s development, so that the greater the number of stressful events, the greater the presence of clinical problems [27]. Studies with adolescents living in at-risk families enrolled in Child Welfare Services (CWS) found complicated risk trajectories in these youngsters, finding that they were particularly susceptible to developing this type of behavioral problem [28,29,30]. That high level of stress may be more harmful to the development of boys and girls in these surroundings than to the adults themselves due, amongst other reasons, to adolescents’ lack of psychosocial maturity to cope with these adversities [28,29,30]. Self-concept studies have shown that those adolescents who have experienced a greater number of significant negative experiences, particularly in areas which are important to them, present more difficulties and fluctuations in self-concept [7].

### 1.2. Parenting and Psychosocial Adjustment in Adolescence

Parental behavior is one of the most influential factors in terms of the development of externalizing and internalizing behaviors of adolescents. A great amount of research has focused on which parenting styles are best at promoting the growth and development of adolescents [31,32]. The typological perspective of parenting is the most widely used, and includes a constellation of parental dimensions such as affection, punishment, dialogue, or indifference [31,32]. This perspective allows a multidimensional approach on parenting [33]. Traditionally, the relations between parenting styles and child adjustment have been studied from the classical two-dimensional orthogonal model of parental socialization. Based on these two dimensions (acceptance/involvement and strictness/imposition), Musitu and García [34] identified four parental socialization styles: authoritative (characterized by the use of high strictness/imposition and high acceptance/involvement); neglectful (low strictness/imposition and low acceptance/involvement); indulgent (low strictness/imposition and high acceptance/involvement); and authoritarian (high strictness/imposition and low acceptance/involvement). This typology emerges from a contextual [31] and situational [35] approach to parenting.

The relation between parenting styles and adolescent adjustment has a great deal of empirical evidence. In general, findings from previous studies have shown better adolescent outcomes with authoritative parents and poorer with neglectful parents; while the authoritarian and indulgent styles have occupied an intermediate position [36,37,38,39]. In general, the available empirical data show adolescents from authoritative families perform better academically [40] are more optimistic [41], make better use of adaptive strategies [36], have less drug use [38], higher self-concept [42], more behavioral regulation [43] and are more resilient [44] than adolescents from families with other parenting styles. Additionally, this relationship remains invariant with regard to the sex of the participants in previous studies. [36,45,46].

Although in general terms the positive effects of the authoritative style and the negative effects of the neglectful style have been widely documented, some studies have shown variations in the effects of the different styles depending on the type of behavioral problem analyzed. In relation to externalizing problems, the review by Marcone, Affuso, and Borrone [43] showed that the most positive style for promoting external adolescent adjustment is the authoritative, and the most harmful is the authoritarian style. This same review did not find such clear-cut results in relation to the indulgent (sometimes associated with positive effects) and neglectful styles (sometimes associated with negative effect styles). These authors concluded that parental control has been linked to beneficial effects if it is exerted as supervision, behavioral control, or guide; in contrast, psychological, coercive, or restrictive control is associated with externalizing behaviors.

There is less research into internalizing problems. The systematic review by Rose, Roman, Mwaba and Ismail [47] of the 2012–2015 period only found five studies focusing specifically on parenting styles and their relation to internalization (articles with children over the age of 12 years and which had a medical focus). These authors found that the lowest levels of internalizing problems were found in adolescents with authoritative parents, and the uninvolved parenting style (neglectful) was associated to the highest level of internalizing problems. In turn, they also found that hostile and punitive parenting was identified as a risk factor for internalization problems in several studies. Despite these results, the systematic review performed by Yap, Pilkington, Ryan, and Jorm [48] showed that the empirical evidence of the relation between authoritarian, authoritative, and indulgent styles and internalizing problems is weak and remains equivocal. The authors concluded that more empirical evidence is needed about the role of parenting styles in belonging to internalizing problems, because other significant factors have been identified for these types of behavior problems (temperamental response styles and emotion regulation strategies) [49].

The association between parenting and behavior problems seems to vary when co-occurrence of internalizing and externalizing problems is controlled. Likewise, Lorence et al. [50] found that the association between parenting and externalizing problems remains invariant once this co-occurrence is controlled, but not for internalizing problems. These authors found that the effect of parenting styles on internal adjustment disappeared while the influence of other variables, as the emotional impact of stressful life events accumulation, is maintained.

Finally, the role of a parenting style varies according to the socio-cultural context [51,52,53,54,55]. Some studies in European or Latin American countries support the indulgent style as the optimum parenting style [56,57,58,59,60,61]. Parenting practices based on acceptance, support, and reasoned communication promote adolescent adjustment regardless of the cultural context, although according to García and Gracia [59], this is particularly beneficial in collectivist cultures, where cooperation and egalitarian relations are emphasized [58,62]. However, findings about the effects of parental control are more controversial when comparing different cultures [55,59,63,64] and specific social environments [65,66,67]. For all of the above, the results on parenting should be understood in the socio-cultural context (country) in which these are studied.

### 1.3. Objectives

This research is proposed to add knowledge to the relationships between mothers’ parenting styles and adolescent adjustment in Spain. Specifically, the objectives of this study were the following:

- To identify profiles of adolescents according to their behavioral problems.
- To explore the individual, family, and social characteristics of these profiles.
- To analyze the potential role of parenting styles in belonging to adolescents’ profiles.

## 2. Methods

### 2.1. Participants

A total of 449 adolescents (211 females and 238 males; *M* = 13.65 years, *SD* = 1.89) from southern Spain participated in this research. The sample was split into two groups: 223 adolescents living in families declared at-risk and enrolled in Child Welfare Services (CWS), and 226 adolescents living in families not receiving intervention (community adolescents). Both groups were similar in age (*t*(447) = −0.250, *p* = 0.803) and sex (χ^2^(1, *N* = 449) = 3.95, *p* = 0.208).

In regard to the families of the participants, the average number of children under 18 in these homes was 2.11 (*SD* = 0.95), and 29.9% were single-parent families (100% single-mother families). The fathers’ mean age was 44.94 years *(SD* = 6.60), the majority had completed primary education (31.3%) or had finished secondary education (29.9%), and 89% of the fathers were working. Regarding the mothers, the mean age was 42.15 years (*SD* = 6.86), and 21.7% had not completed primary education, 37.6% had completed primary education, 25.5% had finished secondary education, and 15.2% had a university degree. As for employment, 62.4% of the mothers were working at the time.

### 2.2. Measures

*Socio-demographic profile*: We developed a questionnaire to collect socio-demographic information about the adolescent (age, sex, and educational level), family (structure of family) and parents (age, sex, educational level, and employment situation).

A short version of the *Inventory of Stressful Life Events* (ISLE, [68]) was administered to adolescents. Fifteen items from the original 29 negative or potentially problematic events were included in this study (situations not directly controllable by the adolescent have been chosen), covering stressful experiences at individual (“severe illness”, “sexual abuse”), family (“family member’s addiction”, “move”, “parental divorce”, “parental conflicts”, “new couple”, “economic hardship”, “death of family members”, “illness of family members”, “a family member moves/leaves home), and peer-related levels (“isolation”, “couple breakup”, “couple betrayal”, “fight with friends”). The occurrence of these kind of events in the previous five years was rated using a dichotomous scale (0 = *no*, 1 = *yes*). The calculation of the accumulated score of SLEs refers to the total number of stressful life events experienced by the subject.

*Parental Socialization Scale for Adolescents* (ESPA29, [34]). This instrument assesses parental socialization strategies in 29 situations of everyday family life: 13 compliance situations (e.g., “a teacher calls your mother and tells her that you are behaving well in class”) and 16 non-compliance situations (e.g., “you arrive home late”). In each of the compliance situations, the adolescent had to rate the parenting practices of Affection (“my mother shows affection”) and Indifference (“my mother seems indifferent”), and in each of the non-compliance situations, the adolescent had to rate the parenting reactions on Dialogue (“my mother talks to me”), Detachment (“my mother doesn’t tell me anything”), Verbal Scolding (“my mother scolds me”), Physical Punishment (“my mother spanks me”), and Revoking Privileges (“my mother takes something away from me”). ESPA29 consists of 116 items ranging from 1 (*never*) to 4 (*always*), providing us with two parenting dimensions: (1) the Acceptance/Involvement dimension, the result of averaging the responses on Affection, Dialogue, Indifference, and Detachment (the last two subscales are reverse coded); and (2) the Strictness/Imposition dimension, by averaging the responses on Verbal Scolding, Physical Punishment, and Revoking Privileges. According to the ESPA29 original authors [34], after dichotomizing the total sample on acceptance/involvement and strictness/imposition, and examining the two parenting dimensions simultaneously, four parenting styles emerged: Authoritative (above the 50th percentile on both dimensions), Authoritarian (above the 50th percentile on strictness/imposition but below the 50th percentile on acceptance/involvement), Indulgent (above the 50th percentile on acceptance/involvement but below the 50th percentile on strictness/imposition) and Neglectful (below the 50th percentile in both dimensions). This study is focused exclusively on mothers’ parenting styles (main caregiver according to adolescents).

*Youth Self-Report* (YSR, [18]) assesses behavior problems in adolescence. YSR consists of 112 items with response options ranging from 0 (*not true*) to 2 (*very true*), which measure eight behavior problems: Withdrawn (e.g., “I would rather be alone than with others”), Somatic complaints (e.g., “I feel dizzy or lightheaded”), Anxiety and depression (e.g., “I cry a lot”), Social problems (e.g., “I’m too dependent on adults”), Thought problems (e.g., “I see things that other people think aren’t there”), Attention problems (e.g., “I feel confused or in a fog”), Aggressive behavior (e.g., “I destroy things belonging to others”), and Rule-breaking behaviors (e.g., “I steal from places other than home”). The first three subscales are related to Internalizing problems, whereas the last two correspond to Externalizing problems. Cronbach’s alphas in this study were α = 0.79 for internalizing problems and α = 0.86 for externalizing problems.

*Multidimensional Self-Concept Scale* (AF5, [69]). This scale considers that self-concept has different, but related, components. This instrument consists of 30 items with a five-point response scale (ranging from 1 “complete disagreement” to 5 “complete agreement”), which is designed to measure five self-concept dimensions: Academic, Social, Emotional, Family, and Physical. Cronbach’s alpha in this study for each dimension was: Academic, 0.82, Social, 0.70, Emotional, 0.77, Family, 0.80, and Physical, 0.78.

### 2.3. Procedure

Adolescents from at-risk families were selected by CWS professionals according to the following inclusion criteria: (1) adolescents between 11–17 years old, without a diagnosed mental health condition; and (2) living in a family being assessed by CWS professionals at a medium level of psychosocial risk. Community adolescents were selected based on a random sample stratified by conglomerates, bearing in mind (in addition to age and sex) the type of school. Fourteen schools were chosen randomly in southern Spain, and the selection of participants was also random between students per academic course of each school. Adolescents from both samples were living in the same neighborhoods.

All the subjects participated in the study voluntarily, after signing an informed consent form in accordance with the Declaration of Helsinki. The aims of the research project were explained, and all the participants were assured that their anonymity would be protected. Ethics approval was obtained from the ethics committee of the Andalusian Health Services (code 22/0509). No monetary incentives were offered.

### 2.4. Data Analyses

Statistical analyses were conducted by using *IBM SPSS* 20 software for Windows [70]. For the univariate analyses, the descriptive statistics were presented through the means, standard deviations, and the minimum and maximum values (Min–Max) of the quantitative variables, thereby describing their central tendency, variability, and range. For the bivariate analyses, *t*-tests for two independent samples were computed, using Student’s *t*-statistic.

For the multivariate analyses, a series of cluster analyses were computed in order to describe the joint variability of the sample with respect to behavior problems. Cluster analysis is a multivariate classification technique that identifies groups of individuals defined by similarities on multiple dimensions, so that members of the resulting groups are as similar as possible to others within their group (high within-group homogeneity) and as different as possible to those in the other groups (low between-group homogeneity) [33]. Prior to clustering, all the selected measures were z-standardized to equate the variables. Although this statistical technique is very robust if assumptions for parametric statistics are violated, following Pérez [71], both the presence of influential extreme cases and the existence of linearity problems were examined before computing the analysis. Initial groupings were obtained using hierarchical cluster analysis with squared Euclidean distance, and the nearest-neighbor method was used as the measure of linkage. The best solution (number of clusters) was determined by examining both the agglomeration schedule and the dendogram. Then, the centroids of these initial clusters were submitted to an iterative clustering procedure (K-means cluster analysis) to refine final cluster membership. Once the cluster dimensions had been determined through confirmatory K-means clustering, the quality of the solution was checked by analyzing atypical subjects, calculating the *t*-distribution of the sample, comparing standardized residuals for all the variables selected, and checking for outliers. To analyze the differences between clusters, post hoc ANOVAs were computed with the selected psychosocial variables.

Finally, multinomial logistic models were performed, taking into consideration deviance distribution to calculate goodness-of-fit, as well as the rate of correct classification of the observed and predicted subjects of the resulting models. Nagelkerke’s pseudo-R^2^ statistic was used to assess the resulting models’ degree of explanation. After creating the models and satisfactory confirming its viability, the meaning and direction of the coefficients using the Wald statistic and odds ratios (OR) were examined.

## 3. Results

### 3.1. Characteristics of Adolescents: Parenting Styles, Stressful Life Events, Behavior Problems, and Self-Concept

The results of the descriptive analyses of the individual, family, and social aspects evaluated in the study are presented in Table 1. It shows that parenting styles were homogeneously distributed, with the Neglectful style being the most frequently reported and Indulgent being the least. On the other hand, adjustment problems obtained a similar average with moderate variability in their scores. In addition, the analyses focusing on the dimension of self-concept reflected that the dimension referring to the family presented the highest average score, but also the lowest academic score. To conclude the descriptive analyses, adolescents had experienced an accumulation of four SLE over the previous five years.

### 3.2. Two-Step Cluster Analysis: Adjustment Profiles of Adolescents

To accomplish the first aim of this study—to identify adolescent profiles according to externalizing and internalizing problems—two-step cluster analysis was computed. The box and whiskers plots were examined for each variable, and the calculation of the Mahalanobis distance did not reveal the existence of either univariate or multivariate extreme cases, respectively. The bivariate correlation indices between the internal and external adjustment problems (*r* = 0.39) did not exceed the value *r* = 0.80, indicating that there were no collinearity problems.

After computing a hierarchical agglomerative cluster analysis, and repeating the procedure with different clustering methods (between-groups linkage, furthest neighbor, and Ward’s method), the visual examination of each dendogram revealed the existence of three initial clusters. To confirm this solution, a K-means cluster analysis was performed requesting the definition of three groups. Iteration history showed that convergence (and thus the absence of center changes in each cluster) was achieved in the 10th iteration.

**Figure 1:**
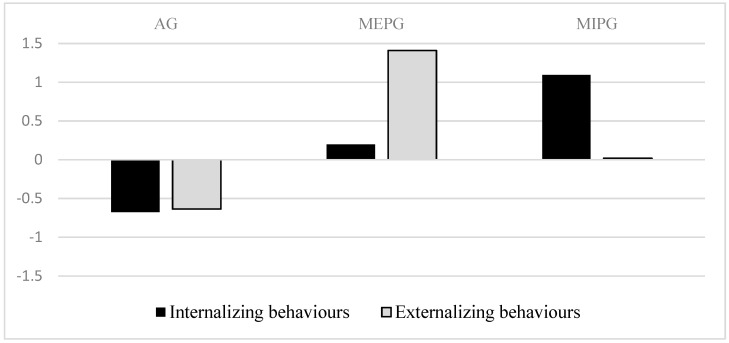
Conglomerate centers offered in typified scores.

Table 2 contains the results of the means in each cluster of behavior problems and an analysis of variance between the conglomerates. Furthermore, Figure 1 shows the centers of the clusters resulting from the analysis.

As Table 2 shows, the three groups were formed differentially depending on both the internal and external maladjustment dimensions. The first cluster (AG; Adjusted Group) differed significantly from the other two, indicating lower scores of internalizing and externalizing problems. The second cluster (MEPG; Maladjustment with Externalizing Problems Group) was characterized by medium scores in internalizing problems and higher scores in externalizing problems. The third cluster (MIPG; Maladjustment with Internalizing Problems Group) revealed high scores of internalizing problems, and medium scores of externalizing problems (see Figure 1).

To complete the analysis, we computed bivariate tests including the three resulting clusters and some individual, family, and social variables (see Table 3). First, we observed that adolescents were not significantly distributed according to sex (χ^2^ = 3.41, *p* = 0.182). As Table 3 shows, adolescents from the AG tended to be community and experience authoritative and indulgent parenting styles, high levels of self-concept and a low accumulation of SLE, and did not perceive authoritarian and neglectful styles. In turn, the MEPG consisted mainly of at-risk adolescents supported by CWS with low self-concept (barring the social one) and a greater accumulation of SLEs. Regarding parenting styles, the adolescents were characterized as experiencing a neglectful parenting style if they did not perceive authoritative and indulgent styles. Lastly, the MIPG cluster was not defined by adolescent background and parenting styles; however, their social, emotional, and physical self-concept tended to be low compared to the other groups, and was characterized by an accumulation of SLEs similar to the MEPG and superior to the AG.

### 3.3. Parenting Styles and Group Membership

Multinomial logistic regression analyses were performed to analyze the potential role of parenting styles in adolescent’s belonging to each cluster. The resulting models estimates the factors associated with the probability that an adolescent from the maladjusted groups will become part of the AG, that is, the one characterized by better indicators in terms of internalizing and externalizing problems. The first important indicator that allows us to ascertain the correspondence of the models with the data is the goodness-of-fit, which is measured through the deviance (Authoritative Model, χ^2^ (46) = 43.86, *p* = 0.562; Indulgent Model, χ^2^ (44) = 39.17, *p* = 0.678; Authoritarian Model, χ^2^ (48) = 42.74, *p* = 0.688; and Neglectful Model, χ^2^ (46) = 38.87, *p* = 0.763), concluding that the models were adequate. Final model likelihood ratio tests (−2LL) were statistically different from the initial ones for all models (Authoritative Model, χ^2^ (4) = 43.54, *p* < 0.001; Indulgent Model, χ^2^ (4) = 46.41, *p* < 0.001; Authoritarian Model, χ^2^ (4) = 38.10, *p* < 0.001; and Neglectful Model, χ^2^ (4) = 50.64, *p* < 0.001). Thus, we proceeded to interpret the models by examining the OR scores, which allowed us to establish whether each selected variable constituted, on its own, an element that increased the probability of belonging to the reference group.

The factors associated with adjustment problems, taking the AG as reference, are presented in Table 4. Specifically, the Authoritative Model explained 10.86% of the variance of the scores, and correctly predicted 53.09% of the subjects belonging to its reference group. As Table 4 shows, the analysis of the OR values of the MEPG showed that the presence of the authoritative style increased the probability that an adolescent would become part of this AG by 77%. This relationship was not observed in the MPIG. Likewise, for each increase in an SLE, the probability that an adolescent belong to the MEPG or MIPG increased by 32% and 22%, respectively.

The Indulgent Model explained 11.54% of the variance and correctly predicted 52.17% of the subjects belonging to its reference group. As Table 5 shows, the analysis of OR values showed that the presence of the indulgent style increased the probability that an adolescent from the MEPG would become part of the AG by 72%, and that an adolescent from the MIPG would become part of the AG by 43%. In turn, for each increase in an SLE, the probability that an adolescent belonged to the MEPG or MIPG, increased by 31% and 21%, respectively.

The Authoritarian Model explained 9.56% of the variance and correctly predicted 51.49% of the subjects belonging to its reference group. As Table 6 shows, the analysis of the OR values showed that the presence of the authoritarian style increased the probability that an adolescent from the AG would become part of the MEPG or MIPG by 58% and 53%, respectively, although the p-values did not report statistical significance. In addition, for each increase in SLE, the MEPG and MIPG increased by 31% and 21%, respectively.

Lastly, the Neglectful Model explained 12.53% of the variance and correctly predicted 53.55% of the subjects belonging to its reference group (AG). As Table 7 shows, the analysis of OR values showed that the presence of the neglectful style increased the probability that an adolescent from AG would become part of the group with externalizing problems (MEPG) by 190%, while no statistical significance was observed in the group with internalizing problems (MIPG). Likewise, for each increase in an SLE, the probability that an adolescent belong to the MEPG and MIPG, increased by 34% and 22% respectively.

## 4. Discussion

Within the framework of a broader study analyzing adolescence from a global and positive perspective, the central objective of this work was to analyze the differential effects of parenting styles on adolescent maladjustment in Spain. Specifically, different profiles of adolescents have been identified considering internal and external adjustment problems together, exploring the individual, family, and social attributes that characterize these profiles, and studying the potential role of parenting styles and the accumulation of stressful events in belonging to these profiles.

While the normative changes that take place in adolescence are conducive to the emergence of adjustment problems [12,13,14,15], not all adolescents experience them in the same way. In this sense, the analyses related to the first objective of the study showed the existence of three different adolescent profiles depending on the prevalence of the type of adjustment problems. Specifically, while the first group (AG) of adolescents was characterized by low levels of both internal and external maladjustment, the analyses carried out showed two other groups with medium–high levels of adjustment problems. The second group (MEPG) was characterized by moderate levels of internal maladjustment and high external adjustment problems, while the third group (MIPG) had moderate levels of external maladjustment and high levels of internal maladjustment. These results underline the importance of studying together both internal and external adjustment problems in adolescence, as they often co-occur in adolescence [9,22].

The second objective of this study was to characterize each cluster by variables on the individual (sex, age, self-concept, stressful life events), family (parenting styles), and social level (community or CWS users). On the individual level, we did not found differences in sex between clusters. Although studies analyzing how internal and external adjustment problems are related to sex have tended to identify the former with girls [16,17] and the latter with boys [6,17,18,19], the relevance of this socio-demographic dimension seems to be disappear when taking into account the co-occurrence of adjustment problems.

Self-concept is another of the individual dimensions that has traditionally been considered as an indicator of the presence of behavioral problems [8]. In the present study, adolescents from different clusters scored differently in self-concept, with AG scoring better than the maladjusted (MEPG and MPIG) in most of the dimensions comprising self-concept. In addition, these results reveal the presence of a complex profile in adolescents with behavioral problems. On the one hand, the members of the MEPG had a negative perception of their family and academic self-concept. These dimensions refer to the two most relevant developmental contexts for children (family and school) where, amongst other issues, they have to comply with rules of behavior. Thus, the adolescents from the MEPG, which were characterized by problems in complying with established norms [12,13], defined themselves more negatively due to their experience in these contexts. In turn, the members of the group characterized especially by internalizing problems (MIPG) stood out due to their low social and emotional self-concept. Problems of loneliness, anxiety, and depression present in boys and girls with these difficulties [14,15] may interfere in their self-description and their self-assessment in these important developmental areas.

We observed differences in the distribution of parenting styles on the three adjustment profiles. The group of adjusted adolescents (AG) was characterized by indulgent and authoritative parenting styles, while those in the MEPG perceived a more neglectful style. These results follow the line of previous studies that stress the importance of the family environment, and more specifically, the type of parent–child interaction and how this affects adolescent adjustment [36,37]. In addition, while the effects of authoritative and neglectful styles on adolescent adjustment have been widely recognized in previous research [36,37,38,39], this paper also highlights the positive role of the indulgent style in defining the group of adjusted adolescents (AG). In contrast to the studies in which the indulgent style is placed in an intermediate position, the results of this work demonstrate that this parental style is related to the absence of adjustment problems. A possible explanation for this is the socio-cultural environment in which this study was performed. Compared to other more individualistic socio-cultural environments, Spain is considered a country with a collectivist-type culture, and previous studies have shown that this style appears to be particularly favorable for adolescent adjustment [56,57,58,59,61].

In the social sphere, the adjusted group (AG) consisted mainly of adolescents from the community sample, while the MEPG was made up of users of CWS, and the MIPG comprises adolescents from both subsamples to equal extent. Thus, those adolescents who break rules and have less self-control and greater aggressiveness tend to be detected and attended by CWS to a greater extent than those who showed internal adjustment problems (loneliness, depression, anxiety...). However, adolescents from both MPEG and MIPG were characterized by a high accumulation of stressful life events around five negative events that were especially relevant in the lives of these boys and girls compared to the adjusted group (AG). The results regarding the accumulation of stressful life events follow the line found in other studies carried out both in community samples [23,24,25,26] and with CWS users [28,29].

As for the last objective of the study, and due to parental behavior being an important predictor of adolescent maladjustment, we explored whether parenting styles could explain the belonging to the groups. We also took into consideration the accumulation of stressful life events as an explanatory dimension. As a result, this study confirms the accumulative hypothesis of risk and its impact on adolescent development [27], since in all of the computed analyses, a greater accumulation of stressful events predicted a change from the AG to the MIPG or MEPG. In turn, less experience of such stressful circumstances increased the likelihood of adolescents with maladjustment profiles (MIPG or MEPG) belonging to the AG. Regarding parenting styles, MEPG adolescents benefit to the same degree from authoritative or indulgent parenting styles, because a greater presence of these styles meant they were more likely to pass from this profile to a more adjusted one. In contrast, a greater degree of neglect increased the likelihood of moving from the adjusted group (AG) to the MEPG. These results follow the line of previous studies, which point, in general terms, to the positive effects of the authoritative style and the negative effects of the neglectful style (e.g., [43]), plus that the indulgent style has a similar influence to the authoritative style for this type of adjustment profile. On the other hand, belonging to the MIPG was not significantly related to any of the parenting styles. This result is similar to the conclusions of the review by Yap et al. [48], where they pointed out that empirical evidence regarding the relationship between parenting styles and internalizing problems is fragile and equivocal. The indulgent style is the only one that seems to be able to explain the internal adjustment problems. It is not surprising that a style centered on affection and communication has some impact on the development of internal adolescent adjustment [56,57,58,59,61]. However, as mentioned above, there may be other personal factors with greater predictive power concerning the adolescent [49], as in the case of the accumulation of stressful events [50].

This study has a number of limitations, amongst which is that the information has been obtained from a single informant. In addition, information has only been collected about the mother’s parenting style, ignoring the role of fathers in adolescent adjustment problems. Having data from different members of the family system and about the parenting styles of both parents would have provided a clearer picture of the problems surrounding these adolescents, as well as how their families functioned. Additionally, it would have been interesting to examine the role of others personal measures of the adolescents (emotional regulation, coping strategies, or locus of control) as well as others related to their social support network (size, composition, or satisfaction). Finally, this study is cross-sectional, and a longitudinal approach would have allowed us to analyze the short-term and long-term effects of parenting styles on adolescent adjustment profiles.

Despite the aforementioned limitations, the main contribution of this study is its potential to improve interventions for families with adolescents. To prevent negative consequences in adulthood, it is essential to develop family interventions that provide adolescents that have problematic profiles with the resources they need to cope with stressful situations [27,28,30]. The results reported and discussed here suggest that it’s important to take into account the risk trajectories of adolescents, as well as their adjustment profiles, to tailor the objectives of family intervention programs. The power of the accumulation of stressful events to explain both internal and external maladjustment profiles makes it essential for interventions to have the empowerment of adolescents as a central objective. This study also highlights the importance of positive parenting practices based on affection, support, communication, and dialogue for adolescent adjustment, in line with previous studies [2]. However, results obtained show that the effects of different parenting styles are not the same for adolescent with different adjustment profiles. Thus, interventions should be tailored to the specific and differing needs of adolescents with internalizing problems and those with a more externalizing maladjustment profile. Specifically, the promotion of authoritative and indulgent parenting styles significantly favors the improvement of the adjustment of adolescents with an external maladjustment profile, but not for those with a maladjustment profile in which internalizing problems predominate.

## 5. Conclusions

The results reported here point out the differing influence of parenting styles on adjustment during adolescence depending on the specific problems (internalizing versus externalizing) experienced by adolescents, and seems to be a central topic to the planning of positive parenting interventions. These results suggest the need to diversify the interventions to fit the specific needs of adolescents with different risk profiles and trajectories (accumulation of stressful life events). Girls and boys with complicated risk trajectories may require individual interventions, with specialists working specifically and directly with them. In addition, this work notes the need to combine this type of intervention with another aimed at promoting positive parenting styles (authoritative and indulgent) in families with adolescents who present a problematic, predominantly externalizing adjustment profile.

## Figures and Tables

**Table 1:** Descriptions of parenting styles, behavior problems, self-concept, and stressful life events.

	Percentage			
Parenting styles				
Authoritative	28.73%			
Indulgent	17.15%			
Authoritarian	22.49%			
Neglectful	30.73%			
	***M***	***SD***	**Minimum**	**Maximum**
Stressful life events (SLE)				
Accumulation SLE	4.19	2.52	0	14
Behavior problems				
Internalizing	0.41	0.22	0	1.26
Externalizing	0.41	0.26	0	1.44
Self-Concept				
Family	4.37	0.68	1.33	5
Social	4.09	0.65	1.67	5
Emotional	3.53	0.77	1	5
Academic	3.31	0.96	1	5
Physical	3.34	0.79	1.17	5

**Table 2:** Descriptive and ANOVA between the conglomerates according to internalizing and externalizing problems.

	AG (49.67%)	MEPG (22.05%)	MIPG (26.95%)	ANOVA	
	*M* (*SD*)	*M* (*SD*)	*M* (*SD*)	*F* (df)	Bonferroni
Internalizing problems	0.26 (0.11)	0.45 (0.19)	0.66 (0.17)	285.95 *** (2,440)	1–2 *** 1–3 *** 2–3 ***
Externalizing problems	0.24 (0.13)	0.77 (0.19)	0.41 (0.17)	406.09 *** (2,440)	1–2 *** 1–3 *** 2–3 ***

*** *p* < 0.001; AG: Adjusted Group, MEPG: Maladjustment with Externalizing Problems Group, MIPG: Maladjustment with Internalizing Problems Group.

**Table 3:** Descriptive and bivariate analyses including the three resulting clusters and different individual, family, and social variables.

	AG (49.67%)	MEPG (22.05%)	MIPG (26.95%)	Crosstabs	
	%(*r_as_*)	%(*r_as_*)	%(*r_as_*)	χ^2^ (df)	*V Cramer*
Background					
Community	30.70% (4.2)	7.67% (−3.8)	12.64% (−1.2)	20.97 *** (2, 441)	0.22 ***
Child Welfare Services	19.64% (−4.2)	14.67% (3.8)	14.67% (1.2)		
Parenting styles					
Authoritative (yes/no)	17.08%/33.71% (2.1/−2.1)	3.87%/17.99% (−2.8/2.8)	8.20%/19.13% (0.2/−0.2)	8.29 * (2, 437)	0.14 *
Indulgent (yes/no)	11.85%/38.95% (3.4/−3.4)	1.59%/20.27% (−2.9/2.9)	3.87%/23.46 (−1.1/1.1)	13.18 ** (2, 437)	0.17 **
Authoritarian (yes/no)	9.11%/41.68% (−2.6/2.6)	6.38%/15.49% (1.6/−1.6)	7.52%/19.82% (1.4/−1.4)	6.60 * (2, 437)	0.12 *
Neglectful (yes/no)	12.76%/38.04% (−2.5/2.5)	10.02%/11.85% (3.7/−3.7)	7.74%/19.58% (−0.6/0.6)	13.96 ** (2, 437)	0.18 **
				ANOVA	
	*M (SD)*	*M (SD)*	*M (SD)*	*F*(df)	*Bonferroni*
Age	13.40 (1.89)	14.25 (1.84)	13.66 (1.92)	5.41 (2, 440)	1–2 * 1–3 2–3
Stressful life events					
Accumulation SLE	3.52 (2.23)	5.25 (2.50)	4.62 (2.68)	19.75 *** (2, 438)	1–2 *** 1–3 *** 2–3
Self-Concept					
Family	4.58 (0.46)	3.99 (0.84)	4.34 (0.70)	30.57 *** (2, 438)	1–2 *** 1–3 ** 2–3 ***
Social	4.16 (0.55)	4.19 (0.73)	3.86 (0.72)	10.66 *** (2, 439)	1–2 1–3 *** 2–3 ***
Emotional	3.79 (0.62)	3.47 (0.76)	3.07 (0.80)	40.51 *** (2, 434)	1–2 ** 1–3 *** 2–3 ***
Academic	3.58 (0.84)	2.61 (0.99)	3.38 (0.88)	39.48 *** (2, 431)	1–2 *** 1–3 2–3 ***
Physical	3.45 (0.74)	3.31 (0.87)	3.17 (0.77)	5.12 ** (2, 438)	1–2 *** 1–3 * 2–3

*r_as_* = adjusted standardized residuals; *p < 0.05, **p < 0.01, ***p < 0.001; AG: Adjusted Group, MEPG: Maladjustment with Externalizing Problems Group, MIPG: Maladjustment with Internalizing Problem Group.

**Table 4:** Multinomial logistic regression model parameters using the AG as a reference and authoritative style as covariate.

Authoritative Model	B	χ^2^ Wald	*p*	OR	OR LB 95%	OR UB 95%
MEPG						
Intercept	−1.81	44.62	<0.001			
SLE	0.28	28.82	<0.001	1.32	1.19	1.46
Authoritative style	−0.88	8.42	0.004	0.23	0.23	0.75
MIPG						
Intercept	−1.36	32.04	<0.001			
SLE	0.20	17.29	<0.001	1.22	1.11	1.34
Authoritative style	−0.19	0.59	0.442	0.83	0.51	1.34

MEPG: Maladjustment with Externalizing Problems Group, MIPG: Maladjustment with Internalizing Problem Group.

**Table 5:** Multinomial logistic regression model parameters using the AG as a reference and indulgent style as covariate.

Indulgent Model	B	χ^2^ Wald	*p*	OR	OR LB 95%	OR UB 95%
MEPG						
Intercept	−1.80	47.81	<0.001			
SLE	0.27	28.39	<0.001	1.31	1.18	1.44
Indulgent style	−1.28	9.86	0.002	0.28	0.12	0.62
MIPG						
Intercept	−1.29	31.53	<0.001			
SLE	0.19	17.24	<0.001	1.21	1.11	1.39
Indulgent style	−0.56	3.63	0.057	0.57	0.32	1.01

MEPG: Maladjustment with Externalizing Problems Group, MIPG: Maladjustment with Internalizing Problem Group.

**Table 6:** Multinomial logistic regression model parameters using the AG as a reference and authoritarian style as covariate.

Authoritarian Model	B	χ^2^ Wald	*p*	OR	OR LB 95%	OR UB 95%
MEPG						
Intercept	−2.09	64.40	<0.001			
SLE	0.27	28.63	<0.001	1.31	1.18	1.44
Authoritarian style	0.46	2.71	0.100	1.58	0.92	2.73
MIPG						
Intercept	−1.48	42.47	<0.001			
SLE	0.19	16.64	<0.001	1.21	1.10	1.32
Authoritarian style	0.42	2.68	0.101	1.53	0.92	2.54

MEPG: Maladjustment with Externalizing Problems Group, MIPG: Maladjustment with Internalizing Problem Group.

**Table 7:** Multinomial logistic regression model parameters using the AG as a reference and neglectful style as covariate.

Neglectful Model	B	χ^2^ Wald	*p*	OR	OR LB 95%	OR UB 95%
MEPG						
Intercept	−2.46	76.83	<0.001			
SLE	0.29	34.90	<0.001	1.34	1.22	1.48
Neglectful style	1.06	18.40	<0.001	2.90	1.78	4.72
MIPG						
Intercept	−1.49	41.98	<0.001			
SLE	0.20	19.85	<0.001	1.22	1.12	1.33
Neglectful style	0.26	1.18	0.276	0.81	0.81	2.08

MEPG: Maladjustment with Externalizing Problems Group, MIPG: Maladjustment with Internalizing Problem Group.
